# Neratinib and Capecitabine for the Treatment of Leptomeningeal Metastases from HER2-Positive Breast Cancer: A Series in the Setting of a Compassionate Program

**DOI:** 10.3390/cancers14051192

**Published:** 2022-02-25

**Authors:** Alessia Pellerino, Riccardo Soffietti, Francesco Bruno, Roberta Manna, Erminia Muscolino, Pierangela Botta, Rosa Palmiero, Roberta Rudà

**Affiliations:** 1 Division of Neuro-Oncology, Department of Neuroscience, University and City of Health and Science Hospital, 10126 Turin, Italy; alessia.pellerino85@gmail.com (A.P.); f.bruno@unito.it (F.B.); emuscolino.neuro.oncologia@gmail.com (E.M.); botta.neuro.oncologia@gmail.com (P.B.); rosapalmiero61@gmail.com (R.P.); 2 Department of Medical, Surgical Sciences and Advanced Technologies GF Ingrassia, University of Catania, 95131 Catania, Italy; robertamanna66@gmail.com; 3 Department of Neurology, Castelfranco Veneto and Treviso Hospital, 31100 Treviso, Italy; rudarob@hotmail.com

**Keywords:** breast cancer, human epidermal growth factor receptor, leptomeningeal metastases, neratinib

## Abstract

**Simple Summary:**

Leptomeningeal metastases represent an unmet need due to the lack of effective therapy and poor survival. The tyrosine kinase inhibitor, neratinib, has demonstrated promising activity against brain metastases from HER2-positive breast cancer, as reported by the TBCRC and NALA trials, thus suggesting a potential activity also in leptomeningeal metastases when associated with capecitabine. The aim of the study was to investigate the efficacy and tolerability of neratinib in association with capecitabine in leptomeningeal metastases from heavily-pretreated breast cancer patients who failed multiple lines of treatment. Primary endpoints were 6-month overall survival and intracranial progression-free survival. Secondary endpoints were the responses assessed by whole CNS MRI performed every 8 weeks, neurological improvement, and safety. We obtained a median overall survival of 10 months, an intracranial progression-free survival of 4 months, neurological improvement and stable disease on an MRI lasting 6.5 months in six patients (60%). These preliminary findings suggest a potential activity of this treatment in LM from HER2-positive breast cancer that needs to be further investigated in larger datasets.

**Abstract:**

Background: Leptomeningeal metastasis is a neurological complication from HER2-positive breast cancer with a poor prognosis and limited treatment options. This study has evaluated the activity of neratinib in association with capecitabine in 10 patients with LM from HER2-positive BC after the failure of multiple lines of treatment, including trastuzumab-based therapy, within a compassionate program, and a comparison was made with a historical control group of 10 patients. Methods: Patients aged ≥ 18 years with histological diagnosis of primary HER2-positive BC, either amplified or mutated, and newly-diagnosed LM were enrolled. Coexistence of BM that has or has not received radiotherapy, as well as prior chemotherapy, hormone therapy, or monoclonal HER2-targeting antibodies or antibody–drug conjugates, were allowed, with the exclusion of lapatinib. Results: Six-months OS was 60% with a median OS of 10 months (95% CI: 2.00–17.0). Three-month intracranial PFS was 60% with a median intracranial PFS of 4.0 months (95% CI: 2.00–6.0). The neurological benefit was observed in 70% of patients with a median duration of neurological response of 6.5 months. The best radiological response was stable disease in 60% of patients. Conclusions: This small series shows that the combination of neratinib and capecitabine is a safe treatment in LM from heavily pretreated HER2-positive BC with clinical efficacy in some patients and is worth investigating in a larger study.

## 1. Introduction

Leptomeningeal metastases (LM) occur when tumor cells infiltrate the leptomeninges of the brain and spinal cord, as well as the cerebrospinal fluid (CSF), with high morbidity and mortality [1]. As patients with breast cancer (BC) have prolonged survival due to the efficacy of new systemic therapies and the diagnosis of LM has improved with MRI, this complication is being diagnosed more frequently. Human epidermal growth factor receptor 2 (HER2) is overexpressed in approximately 15%–20% of patients with BC [2], of whom up to 50% develop brain metastases (BM), and 5% LM alone. Moreover, 43% of patients with BM may progress with secondary LM [3]. In general, LM from HER2-positive BC has a poor median overall survival (OS), ranging from 6.6 months for HER2+/estrogen receptor-positive (ER+) to 11.4 months for HER2+/ER negative BC, respectively [4]. Therefore, LM is an emerging, unmet, and urgent need for new therapeutic options.

Neratinib is a 4-anilino-3-cyano quinoline derivative, which acts as an irreversible tyrosine kinase pan-inhibitor (TKI) of HER1/HER2/HER4 receptor. The covalent binding with the cysteine residue (Cys773 in HER1 and Cys805 in HER2), located in the adenosine triphosphate protein (ATP) binding pocket of the receptor, leads to the inhibition of the receptor kinase activity, as well as the reduction of the cyclin D1 expression, resulting in a block of the cell cycle in the G1/S phase and suppression of cell proliferation [5]. Moreover, neratinib is not a substrate of ATP-binding cassette and multidrug efflux ABCG2 transporters on the blood–brain barrier (BBB), leading to an increased CNS concentration as compared with lapatinib [6]. Encouraging results in asymptomatic BM were reported in the phase 2 trial NEfERT-T, where CNS recurrence was less frequent in patients treated with paclitaxel plus neratinib (8.3%) as compared with patients receiving paclitaxel plus trastuzumab (17.3%) [7]. The phase 2 TBCRC 022 trial has investigated the activity of neratinib alone in pre-treated BM, reporting that 3 out of 40 (8%) patients achieved a partial response (PR). Notably, the CNS response significantly increased when neratinib was associated with capecitabine up to 49% and 33%, as well as median progression-free survival (PFS) to 5.5 months and 3.1 months, in lapatinib-naive and lapatinib pre-treated patients, respectively. Interestingly, three patients with secondary LM were enrolled in cohort 3b of the TBCRC trial, reporting one partial response after 7 months of treatment, one stable disease and one progressive disease after 4 months [8]. Further insights were provided by the randomized phase 3 NALA trial that has confirmed that intracranial objective response rate, PFS and OS were higher in patients with BM treated with neratinib plus capecitabine (26.3%; 7.8 months; 16.4 months) as compared with patients who received lapatinib plus capecitabine (15.4%; 5.5 months; 15.4 months) [9]. Furthermore, neratinib plus capecitabine as compared with lapatinib and capecitabine was also associated with a longer PFS (12.4 months vs. 8.3 months), and lower cumulative incidence of interventions for CNS disease (25.5% vs. 36.0%), and progressive CNS disease (26.2% vs. 41.6%), suggesting that such a combined treatment could be a valuable option in patients with CNS involvement from HER2-positive metastatic BC [10].

Building on these results, the aim of the study was to evaluate the activity of neratinib in association with capecitabine in LM from HER2-positive BC after the failure of multiple lines of treatment, including trastuzumab-based therapy, within a compassionate program.

## 2. Materials and Methods

### 2.1. Eligibility Criteria

Eligible patients had the following characteristics: ≥18 years of age; histological diagnosis of primary HER2-positive BC either amplified or mutated; newly-diagnosed treatment-naive LM defined by either clinical and MRI findings consistent with leptomeningeal spread or by positive CSF cytology [11]; Karnofsky performance status (KPS) ≥60 at the time of diagnosis of LM; stable dose of steroids (dexamethasone of 4 mg daily or less for 7 days prior to initiation of treatment); life expectancy of at least 3 months; no evidence of compromised hematologic, hepatic or renal function on laboratory testing, and HBV/HBC/HIV negative test. There were no specific radiological criteria for eligibility. Coexistence of BM, that has or has not received whole-brain radiotherapy (WBRT) or radiosurgery, as well as systemic chemotherapy or targeted therapy (including docetaxel, vinorelbine, trastuzumab-pertuzumab, T-DM1, and hormone therapy), was allowed.

Patients were excluded if they had a suspected diagnosis of bacterial, fungal or viral meningitis, or a known history of active systemic inflammatory diseases that may mimic leptomeningeal enhanced lesions, or received other anti-HER2 “small molecules” (e.g., lapatinib, pyrotinib, tucatinib), prior or concomitant intrathecal therapy, or other investigational agents.

All patients had to sign an informed consent. The study was conducted according to the guidelines of the Declaration of Helsinki, and the protocol was approved by the local IRB “Comitato Etico Interaziendale—A.O.U. Città della Salute e della Scienza di Torino”. The full study protocol is available in Supplementary Material File S1.

### 2.2. Study Design and Treatment

This was an observational study within a compassionate program of neratinib plus capecitabine in LM from HER2-enriched BC either amplified or mutated. The drugs were administered orally according to the manufacturer’s guidelines (Puma Biotechnology, Inc, and Roche, respectively) and the schedule employed in TBCRC 022 [8] and NALA trials [9]. Patients received neratinib (240 mg daily continuously) with capecitabine (750 mg/m^2^ twice per day for 14 days, then 7 days off) until unacceptable toxicity and/or neurological deterioration. Conversely when patients displayed radiological progression, but still neurological benefit was present, neratinib plus capecitabine were continued. Any adverse event was evaluated using Common Terminology Criteria for Adverse Events (CTCAE version 5.0). Prophylaxis with oral loperamide was used in case of severe diarrhea, and delays or dose adjustments of both capecitabine and neratinib were permitted according to the protocol (see Supplementary Material File S1).

All patients underwent physical and neurological examination, laboratory testing, ECG, echocardiogram, and CT of chest/abdomen/pelvis at baseline. Afterward, physical, and neurological examination and blood testing were performed monthly, while ECG and echocardiogram were performed every 3 months. Brain and spinal MRI with gadolinium were performed at baseline and every 2 months. CSF examination at baseline and during treatment was not mandatory to avoid further discomfort to these patients with advanced disease. Patients underwent CT chest/abdomen/pelvis every 3 months for monitoring systemic disease.

### 2.3. Endpoints

The co-primary endpoints were 6-month OS, which was measured from the date of treatment start to the date of death or last follow-up (whichever occurred first), and intracranial progression-free survival (i-PFS), which was measured from the date of treatment start to the date of first LM progression or death or last follow-up (whichever occurred first). Secondary endpoints were response on whole CNS MRI based on RANO-LANO criteria [1], neurological improvement, as defined as an improvement ≥2 months of at least 2 neurological symptoms and safety. The response on MRI was reviewed independently by 2 neuroradiologists, who were unaware of neurological response and outcome. Moreover, we retrospectively identified in our institutional database a control group of 10 patients with LM with similar characteristics who received intrathecal liposomal cytarabine (Ara-C, n = 3) alone or in association with WBRT (n = 7) and calculated i-PFS and OS to be compared with those of the patients in this series. Clinical data were collected using MedCalc software (version 20.015).

### 2.4. Statistical Analysis

Baseline characteristics of patients included in the analysis are summarized using percentages and frequencies (n, %). Overall survival (OS) and intracranial PFS (i-PFS) were calculated from the date of treatment start to the date of recurrence or death, respectively, or until the last follow-up visit (censoring). Six-month OS and i-PFS rates are reported 95% exact binomial confidence intervals. The distribution of OS and i-PFS are presented using the Kaplan–Meier method with 95% confidence intervals estimated using log(−log) methods. Statistical analyses were conducted using MedCalc^®^ software v20.015. Toxicities are summarized according to the worst grade occurring in each patient.

## 3. Results

### 3.1. Patients

Eleven patients were enrolled at the Division of Neuro-Oncology, Dept. Neuroscience of the University and City of Health and Science Hospital, Turin, Italy, from February 2018 to November 2021. Of these patients, one did not receive therapy due to clinical deterioration. All patients were female, with a median age at the time of the enrollment of 43 years (range 36–69 years; Table 1). Seventy percent of patients (n = 7) had a median KPS of 80 (range 60–90). Nine patients were HER2-positive hormone-receptor-positive (triple positive), of whom the rates of estrogen receptor (ER) and progesterone receptor (PR) positivity were 100% (9 of 9) and 33.3% (3 of 9), respectively, while one patient had HER2-enriched hormone-receptor-negative BC (ER-/PR-/HER2+) with an L755S mutation in the kinase domain. Five patients remained on endocrine therapy (letrozole or fulvestrant) concurrently with neratinib and capecitabine. Ninety percent of patients (n = 9) had coexisting extracranial disease, of whom the most common sites were bone (n = 6) and lung (n = 3). All patients were heavily pretreated with a median of three prior systemic therapies (range 2–5). Overall, 60% of patients (n = 6) with BM received radiotherapy. As for the two patients with BM treated with surgery, one patient received adjuvant conformal radiotherapy, and one was treated with adjuvant stereotactic radiosurgery; the other four patients received WBRT alone. The median time between the radiotherapy for BM and the initiation of neratinib plus capecitabine was 43 months (range 23–91). LM occurred after a median time since the initial diagnosis of primary BC of 45 months (range 11–166). On MRI, cranial involvement was seen in 7 out of 10 (70%) patients, spinal involvement in one (10%) patient, and both cranial and spinal involvement in two patients (20%). All patients were symptomatic for LM, with a median number of four neurological symptoms (range 2–7) at baseline. The most common neurological symptoms were headache (60%), nausea and vomiting (40%), diplopia and oculomotor nerve palsies (40%), radicular and back/neck pain (40%) (see Table A1 in Appendix A). All patients were receiving a median dose of 8 mg daily of dexamethasone (range 4–8) to control neurological symptoms. Three patients (30%) only underwent lumbar puncture at baseline: CSF cytology was positive for atypical/suspicious cells in two patients, and for neoplastic cells in one patient. Table 1 shows the baseline characteristics of patients receiving neratinib plus capecitabine and those of the control group.

### 3.2. Efficacy

Patients received a total of 72 cycles of neratinib plus capecitabine with a median of 5.5 cycles per patient (range 2–19). In the control group, a total of 38 cycles of intrathecal Ara-C were administered with a median of four cycles per patient (range 1–9), while WBRT (30Gy/10 fractions) was associated with seven patients.

**Figure 1:**
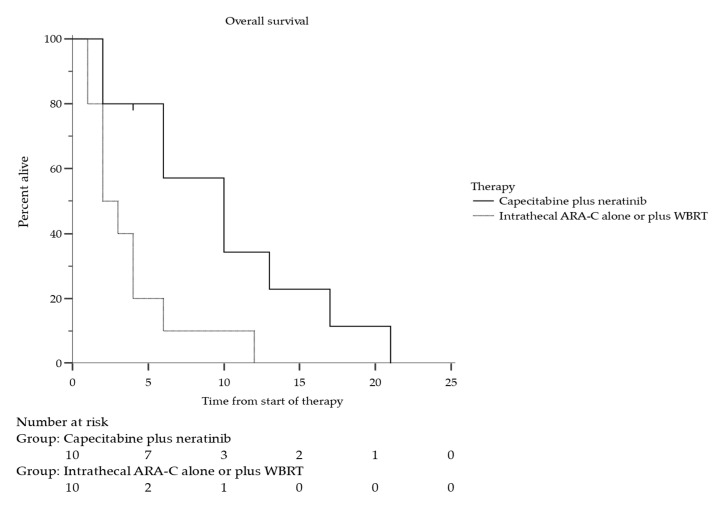
Overall survival in 10 patients with LM from HER2-positive BC treated with neratinib plus capecitabine compared with 10 patients (control group) treated with intrathecal Ara-C alone or in association with WBRT. Median overall survival was 10 months (95% CI: 2.0–17.0 months) for neratinib plus capecitabine treatment group, and 2 months (95% CI: 1.0–4.0 months) for control group (HR 0.21; *p* = 0.0081).

Six out of 10 patients (60%) were alive at 6 months after the initiation of therapy (6-month OS proportion = 0.57; 95% CI: 0.41–0.73) in the neratinib plus capecitabine group, and three patients (30%) were alive at 12 months (12-month OS proportion = 0.23; 95% CI: 0.09–0.37; Table 2). There were 9 deaths out of 10 patients (90%). The median OS was 10 months (95% CI: 2.00–17.0; Figure 1). In the control group, 1 out of 10 patients (10%) was alive at 6 months after the initiation of therapy (6-month OS proportion = 0.10; 95% CI: 0.01–0.19; HR 0.21; *p* = 0.0081), and the median OS was 2 months (95% CI: 1.0–4.0 months).

**Figure 2:**
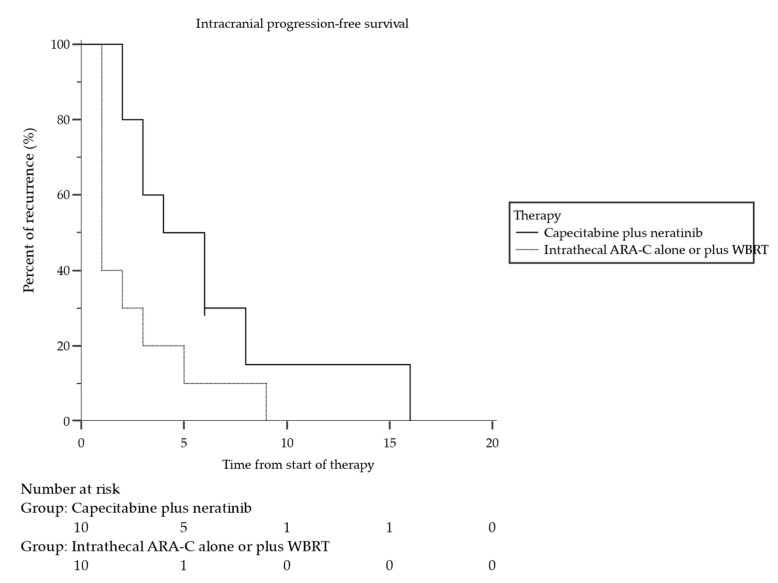
Intracranial progression-free survival (i-PFS) in 10 patients with LM from HER2-positive BC treated with neratinib plus capecitabine compared with 10 patients (control group) treated with intrathecal Ara-C alone or in association with WBRT. Median i-PFS was 4 months (95% CI: 2.0–6.0 months) for neratinib plus capecitabine treatment group, and 1 months (95% CI: 1.0–3.0) for control group (HR 3.1; *p* = 0.04).

All patients had CNS recurrence: in the neratinib and capecitabine group, 8 out of 10 patients (80%) had a leptomeningeal progression, while 2 out of 10 (20%) had a brain progression, while all 10 patients in the control group had a leptomeningeal progression. Four patients (40%) had intracranial progression following 3 months of treatment with neratinib plus capecitabine (3-month i-PFS proportion = 0.60; 95% CI: 0.44–0.76), four patients (40%) after 6 months (6-month i-PFS proportion = 0.30; 95% CI: 0.15–0.046), and two patients (20%) after 9 months of treatment (9 month i-PFS = 0.15; 95% CI 0.03–0.28; Table 2). Median intracranial PFS was 4.0 months (95% CI: 2.00–6.0; Figure 2). In the control group, eight patients (80%) had intracranial progression following 3 months of treatment (3-month i-PFS proportion = 0.20; 95% CI: 0.08–0.33), and other two patients (20%) had leptomeningeal relapse at 6 months, with a median intracranial PFS of 1.0 months (95% CI: 1.0–3.0; HR 3.1; *p* = 0.04), Figure 2).

We did not observe complete or partial responses on MRI either after neratinib plus capecitabine or in the control group. A total of 6 out of 10 patients (60%) and 3 out of 10 patients (30%) achieved stable disease after neratinib plus capecitabine and in the control group, respectively, while 4 out of 10 (40%) and 7 out of 10 (70%) had progressive disease after neratinib plus capecitabine and in the control group, respectively. As for the extracranial radiological response, most of the patients achieved stable disease (6 out of 10, 60%), two patients (20%) had a complete response, one patient (10%) had a partial response, and one patient (10%) had progressive disease following after neratinib plus capecitabine treatment. When comparing intra- and extracranial best responses, among the six patients who achieved stable disease intracranially, two patients reported a complete response, three reported a stable disease, and one patient a progressive disease, respectively, extracranially. Among the four patients who achieved a progressive disease intracranially, one patient reported a partial response, and three patients had a stable disease extracranially (Table A3 in Appendix A).

At the time of death or last follow-up, seven patients (70%) had CNS-only progression, and three (30%) had a recurrence both in the CNS and extracranially. Data on the response of the systemic disease to treatment were available only for patients treated with neratinib plus capecitabine.

As for neurological improvement following neratinib plus capecitabine, three patients (30%) reported a significant improvement of neurological symptoms, while four patients (40%) remained stable with an overall clinical benefit of 70%. Three patients (30%) had a worsening of neurological symptoms. In the control group, most of the patients (70%) reported a neurological deterioration, and only three patients (30%) reported a clinical stabilization of neurological symptoms lasting 1 month (See Table 2). The median number of neurological symptoms after the treatment with neratinib plus capecitabine was 3.5 (range 1–11): of note, patients who achieved a clinical benefit had a significant reduction of the number of neurological symptoms from four before the initiation of neratinib plus capecitabine to two following treatment (See Table A2 in Appendix A). The median duration of neurological response was 6.5 months (range 1–19). Thirty percent of patients (3 out of 10) were able to reduce the daily dose of steroids (≤2 mg/day), while 7 out of 10 patients (70%) needed to keep the same dose. Data of CSF from serial lumbar punctures were available in three patients who received neratinib plus capecitabine: clearance of CSF cytology was observed in one patient only following 2 months of treatment and was associated with an improvement of neurological symptoms (see Patient ID 3 on Table A1 and Table A2 on Appendix A).

### 3.3. Tolerability

In the neratinib plus capecitabine group, 8 of 10 patients (80%) had one or more adverse events that were considered treatment-related, and three patients (30%) had severe adverse events (≥3 according to CTCAE) related to the treatment. No patient had discontinued treatment due to unacceptable toxicity. Fatigue and diarrhea were the most frequent adverse events in 50% and 40% of patients, respectively. Diarrhea typically presented within 48 h of treatment, and peaks within the first week: adequate prophylaxis with loperamide resolved diarrhea over 2 weeks in all patients. No further treatments or dose adjustments were needed to manage gastrointestinal or hepatic side effects (Table 3).

## 4. Discussion

With the extensive use of monoclonal HER2-targeting antibodies, such as trastuzumab and pertuzumab, and antibody–drug conjugates, such as trastuzumab emtansine and trastuzumab deruxtecan, the systemic disease control and survival of patients with HER2-positive BC have dramatically improved [11,12,13]. However, due to the development of resistance to anti-HER2 therapy, coupled with a poor penetration of monoclonal antibodies through the BBB and blood–CSF barrier, an increasing number of patients relapse into the leptomeninges after the failure of multiple lines of treatment [14]. The distinct pathways underlying the invasion of leptomeningeal space, as compared with the brain, as well as the interaction of tumor cells with different microenvironments, seem to be an additional obstacle for finding effective treatments [15]. As LM often is a late neurological complication of heavily pretreated BC occurring in patients with poor performance status and a prognosis of few weeks, the enrollment in clinical trials was not allowed in the past, limiting the knowledge of the activity of many drugs [16].

To our knowledge, this is the first study that has investigated the association of neratinib and capecitabine in LM from HER2-positive BC. Historical series have analyzed the role of intrathecal therapy, including methotrexate (MTX), liposomal cytarabine, and thioTEPA, in LM from mixed primary tumor histologies (including BC), and reported a radiological response in 20%–55% of patients [17,18], and an OS of 3.5–7.6 months [19,20]. As for the neurological benefit following intrathecal treatment, Boogerd et al. reported an improvement in 59% of patients [19], while Grossman et al. did not find any significant neurological advantage, as 75% deteriorated neurologically within 8 weeks after the start of intrathecal therapy [21].

The outcomes of patients in our control group are in line with the results of historical controls and highlight the limited impact of intrathecal therapy alone or in association with radiotherapy in terms of neurological benefit and disease control. However, we highlight that the comparison between patients receiving neratinib plus capecitabine and those of the retrospective control group simply reinforces the suggestion of the existence of a signal of the efficacy of the combination that needs to be fully evaluated in prospective trials. Based on the neurological outcome, radiological response rate, and OS achieved in our study, coupled with the increasing number of targeted therapies to control the extracranial disease, we may argue that intrathecal cytotoxic chemotherapy will not be widely employed in daily clinical practice in the future. Conversely, the intrathecal route could improve the CSF concentration of the monoclonal HER2-targeting antibody trastuzumab. In this regard, it was reported that intrathecal trastuzumab may achieve CSF concentrations 49–420 times higher than when administered intravenously [22]. Bonneau et al. [23] reported a significant clinical improvement in 68.8% of 11 patients, without any concern of tolerability. Likewise, Figura et al. [24] have shown that intrathecal trastuzumab conferred a prolonged 6-month PFS (44%) and 12-month OS (54%) when associated with systemic therapy, as compared with intrathecal MTX/thioTEPA (18% and 10%, respectively). Last, a recent metanalysis on the activity of intrathecal trastuzumab alone (20 patients) or in combination with systemic therapy (37 patients) displayed a clinical improvement in 55.0% of patients, stable disease in 14%, and a median PFS and OS of 5.2 months and 13.2 months, respectively [25]. The results of the Figura study, which are similar to our study, and the data of Zagouri metanalysis, which are slightly better in terms of PFS and OS as compared to those of our cohort, suggest that intrathecal trastuzumab might be effective and safe. However, some concerns may be raised about the feasibility to perform multiple lumbar punctures in heavily pretreated patients with a significant burden of neurological symptoms. First, a lumbar puncture may lead to some discomfort and local pain, especially when multiple attempts are needed in case of scoliosis, severe arthrosis, or back/radicular pain. Second, intrathecal compounds may only penetrate the leptomeninges for 2–3 mm, thus it is preferred in patients with linear leptomeningeal lesions, while the activity on nodular or bulky disease is limited. Moreover, some complications may occur following intrathecal therapy, including aseptic or chemical meningitis, arachnoiditis, and delayed leukoencephalopathy with seizures [26]. The intraventricular route with an Ommaya reservoir was used as an alternative route [27], but the management of the device may be difficult, and careful handling is required to avoid obstruction and infection [26].

Great efforts have been made for improving the ability of targeted therapies to achieve CSF/CNS adequate concentrations and overcome the need to use invasive approaches. In this regard, ultrasounds, that transiently disrupt the BBB, and result in higher penetration of large and polar molecules, are promising and under investigation [28].

Some initial reports of activity of immunotherapy in LM were reported with inferior results in terms of OS as compared with our study. Brastianos et al. [29] conducted a phase 2 trial of ipilimumab and nivolumab in 18 patients with LM from mixed solid primary histologies (5 patients with BC, whose molecular subgroup is unknown) with a 3-month OS of 44% and median OS of 2.9 months (90% CI: 1.6–5.0 months). Pembrolizumab was evaluated in a phase 2 trial with 20 patients with LM, of whom 17 out of 20 from BC (6 patients with HER2-positive BC), with a 3 month-OS of 60%, and a median OS of 3.6 months (90% CI 2.2–5.2 months) [30]. Notably, the median OS for HER2-positive patients was 4.4 months (90% CI, 1–6.8 months), which is inferior to that in our cohort. More in general, checkpoint-inhibitors have shown limited activity in metastatic BC, with the exception of atezolizumab in association with nab-paclitaxel, which in a study conferred a prolonged PFS and OS to patients with advanced triple-negative and PD-L1-positive tumors [31]. However, this study excluded patients with LM.

Patients in our cohort were enrolled in a compassionate program, that allowed them to continue neratinib and capecitabine therapy beyond radiological progression when the patients still were reporting a neurological benefit. Interestingly, the median duration of the neurological benefit was longer (6.5 months) as compared to that with intracranial-PFS (4 months) based on MRI, suggesting that patients may derive a prolonged control of clinical symptoms with an acceptable profile of toxicity, as reported in other metastatic solid tumors [32,33,34].

Our study has several limitations. The small sample size is inherent to the rarity and type of disease and limits the generalization of findings. Furthermore, patients were pretreated with different sequences of therapies for both systemic and CNS disease: this may have interfered with the evaluation of the efficacy of neratinib and capecitabine when delivered as “salvage” therapy for LM. Moreover, the heterogeneity of the cohort in terms of prior CNS and systemic treatments may have unpredictably influenced the molecular divergence between primary solid tumor, brain and leptomeningeal metastases over time. The ideal situation would be to analyze leptomeningeal tissue for the detection of druggable mutations at baseline. However, when dealing with leptomeningeal metastases, to obtain neuropathological tissue for molecular analysis has technical and clinical limitations that make this approach commonly unfeasible in clinical trials. The poor compliance of patients to undergo a lumbar puncture for monitoring response to treatment represents an unsolved issue in the management of LM. Last, the issue of whether neratinib is better than lapatinib when added to capecitabine in women with LM from HER2-positive BC, who are resistant to monoclonal antibody-based therapy, is not clear. In fact, no series on LM from HER2-positive BC who received lapatinib plus capecitabine was reported, except for one case report demonstrating a remarkable clinical and radiological response lasting more than 12 months [35].

The scenario is rapidly evolving after the investigation of tucatinib in the HER2CLIMB trial on HER2+ metastatic BC, which displayed superiority in all the planned outcomes, such as PFS, OS, objective response rate [36] and quality of life [37] including in patients with BM [38]. Recently, Murthy et al. reported the results of a phase 2 TBCRC049 trial focused on 17 patients with LM from HER2-positive BC, who were treated with tucatinib, trastuzumab, and capecitabine: an i-PFS of 6.9 months (95% CI 2.3–13.8) and an OS of 10 months (95% CI 4.1-not reached) were observed [39]. Patients received a median number of only one prior treatment before the start of treatment, which may partly explain the longer i-PFS and OS in comparison with our cohort. Notably, tucatinib and the metabolite ONT-993 were detected in both plasma and CSF [40].

## 5. Conclusions

In this study the treatment of patients with LM from HER2-positive BC with the association of neratinib and capecitabine showed both intra- and extracranial activity, leading to neurological benefit and extended OS in comparison with historical controls. Although the data presented herein are encouraging, our conclusions are necessarily tempered by the small size of the study and should be interpreted as a preliminary suggestion that a signal of efficacy may be present, but future prospective studies are needed to validate these results in larger cohorts of patients. Furthermore, monitoring of the disease response with liquid biopsy of CSF is under investigation in clinical trials and could be of help for a better interpretation of neuroimaging findings.

## Figures and Tables

**Table 1:** Patient’s characteristics at baseline.

	Neratinib Plus	Control Group
	Capecitabine Group	
Factor	N (%)	N (%)
Sex		
Female	10 (100)	10 (100)
Male	0	0
Median age, years (range)	45 (36–59)	45 (35–67)
≤45 years	6 (60)	5 (50)
>46 years	4 (40)	5 (50)
Primary Breast Cancer		
HER2+ ER+ PR+	3 (30)	2 (20)
HER2+ ER+ PR−	6 (60)	6 (20)
HER2+ ER− PR−	1 (10) ^1^	2 (20)
Median KPS (range)	80 (60–90)	70 (60–90)
≤80	7 (70)	8 (80)
≥90	3 (30)	2 (20)
Median time since initial diagnosis of primary BC, months (range)	45 (11–166)	35 (13–58)
≤45 months	5 (50)	7 (70)
>46 months	5 (50)	3 (30)
Systemic disease		
Stable/controlled	6 (60)	4 (40)
Progressive	4 (40)	6 (60)
Site of extracranial disease at the time of LM diagnosis		
Bone	6 (60)	6 (60)
Liver	2 (20)	3 (30)
Lung	3 (30)	2 (20)
Lymph nodes	1 (10)	6 (60)
Skin	1 (10)	0 (0)
Breast	1 (10)	0 (0)
Without extracranial disease	1 (10)	2 (20)
BM at the time of LM diagnosis		
Yes	6 (60)	6 (60)
No	4 (40)	4 (40)
Median number of systemic		
treatments before LM diagnosis (range)	3 (2–5)	3 (2–4)
≤2	4 (40)	5 (50)
≥3	6 (60)	5 (50)
Local therapy for BM before		
LM diagnosis		
Surgery	2 (20)	2 (20)
Radiotherapy	6 (60)	5 (50)
Patterns of LM on MRI		
Cranial LM	7 (70)	5 (50)
Spinal LM	1 (10)	3 (30)
Cranial and spinal LM	2 (20)	2 (20)

^1^ HER2—L755S mutation. ER: estrogen receptor; PR: progesterone receptor; HER2: Human epidermal growth factor receptor 2; BC: breast cancer; LM: leptomeningeal metastases; BM: brain metastases.

**Table 2:** Efficacy of neratinib in association with capecitabine on LM from HER2-positive BC in comparison with control group.

	Neratinib Plus Capecitabine Group	Control Group
	N (%)	N (%)
Overall OS		
At 6 months	7 (70)	1 (10)
At 12 months	3 (30)	0 (0)
Intracranial PFS		
At 3 months	4 (40)	2 (20)
At 6 months	4 (40)	1 (10)
At 9 months	1 (10)	0 (0)
>9 months	1(10)	0 (0)
Best intracranial response ^1^		
Complete response	0	0
Partial response	0	0
Stable disease	6 (60)	3 (30)
Progressive disease	4 (40)	7 (70)
Best extracranial response ^2^		
Complete response	2 (20)	Not applicable
Partial response	1 (10)	
Stable disease	6 (60)	
Progressive disease	1 (10)	
Neurological benefit		
Improvement	3 (30)	0 (0)
Stable	4 (40)	3 (30)
Worsening	3 (30)	7 (70)

^1^ Radiological response was evaluated according RANO/LANO criteria. ^2^ Radiological response of systemic disease was assessed using RECIST criteria. OS: overall survival; PFS: progression-free survival.

**Table 3:** Adverse events from the association of neratinib and capecitabine on LM from HER-2 positive BC.

Adverse Events ^1^	N (%)	Grade 1–2	Grade 3–4
Gastrointestinal disorders Abdominal pain or discomfort Diarrhea Nausea, vomiting Liver function test elevation	2 (20) 4 (40) 3 (20) 1 (10)	2 2 2 1	- 2 1 -
Constitutional Fatigue Weight loss Anorexia	5 (50) 1 (10) 1 (10)	5 1 1	- - -
Skin Hand-foot syndrome	1 (10)	1	-

^1^ Adverse events were evaluated according to CTCAE version 5.0.

## Data Availability

The data presented in this study are available within the article or Supplementary Material.

## Supplementary Materials

The following are available online at https://www.mdpi.com/article/10.3390/cancers14051192/s1, File S1: Study protocol.

## Appendix A

**Table A1:** Neurological symptoms at baseline.

		Patient ID									
General Symptoms	N (%)	1	2	3	4	5	6	7	8	9	10
Headache	6 (60)	X	X	X							
Nausea, vomit	4 (40)										
Seizures	2 (20)										
**Cranial Nerve Impairment**											
Optic neuritis/visual impairment	2 (20)										
Hearing loss	3 (30)										
Facial weakness	1 (10)										
Diplopia/oculomotor nerve palsies	4 (40)										
Trigeminal hypoesthesia	1 (10)										
**Gait Disturbances**											
Sensitive ataxia	1 (10)										
Cerebellar ataxia	2 (20)										
**Cerebellar Symptoms**											
Dysmetria/limb ataxia	4 (40)										
Nystagmus	2 (20)										
Truncal ataxia	1 (10)										
Intention tremor	2 (20)										
Dysarthria/scanning speech	2 (20)										
Vertigo	1 (10)										
**Spinal Symptoms**											
Lower motor neuron weakness	0										
Sensory loss	1 (10)										
Radicular and back/neck pain	4 (40)										
Bladder, bowel, sexual dysfunctions	0										
Total score		4	3	7	7	3	4	3	2	6	4

**Table A2:** Neurological symptoms following treatment with capecitabine and neratinib.

	Patient ID										
General Symptoms	N (%)	1	2	3	4	5	6	7	8	9	10
Headache	4 (40)	X	X								
Nausea, vomit	3 (30)										
Seizures	2 (20)										
**Cranial Nerve Impairment**											
Optic neuritis/visual impairment	2 (20)										
Hearing loss	3 (30)										
Facial weakness	1 (10)										
Diplopia/oculomotor nerve palsies	1 (10)										
Trigeminal hypoesthesia	1 (10)										
**Gait Disturbances**											
Sensitive ataxia	2 (20)										
Cerebellar ataxia	2 (20)										
**Cerebellar Symptoms**											
Dysmetria/limb ataxia	4 (40)										
Nystagmus	2 (20)										
Truncal ataxia	2 (20)										
Intention tremor	2 (20)										
Dysarthria/scanning speech	3 (30)										
Vertigo	1 (10)										
**Spinal Symptoms**											
Lower motor neuron weakness	1 (10)										
Sensory loss	2 (20)										
Radicular and back/neck pain	4 (40)										
Bladder, bowel, sexual dysfunctions	3 (30)										
Total score		4	8	2	11	1	4	3	2	8	1
Overall neurological response		S	P	I	P	I	S	S	S	P	I

I: improvement; S: stabilization; P: progression.

**Table A3:** Concordance between intra- and extracranial best response following treatment with capecitabine plus neratinib in metastatic HER2-positive BC.

Patient	Intracranial Best Response	Extracranial Best Response
1	SD	SD
2	PD	PR
3	SD	CR
4	SD	SD
5	SD	CR
6	PD	SD
7	PD	SD
8	SD	SD
9	PD	SD
10	SD	PD

CR: complete response; PR: partial response; SD: stable disease; PD: progression of disease.
