# Supplementary material for: Neratinib and Capecitabine for the Treatment of Leptomeningeal Metastases from HER2-Positive Breast Cancer: A Series in the Setting of a Compassionate Program
Source: Cancers (Basel). 2022 Feb 25;14(5):1192. doi: 10.3390/cancers14051192 (PMC8909342; doi:10.3390/cancers14051192)
Supplement: Supplementary file 1 [file cancers-14-01192-s001.zip › cancers-1578323-supplementary.pdf]

cancers

MDPI

Study protocol

Prospective study on the efficacy of Neratinib plus capecitabine in patients with leptomeningeal metastases (leptomeningeal carcinomatosis) from HER2 + breast carcinomas

- *INTRODUCTION AND STUDY RATIONALE*

Breast cancer is the most common cause of leptomeningeal metastases with a frequency of 3-5% within solid tumors. Similar to parenchymal brain metastases, the risk of leptomeningeal metastases (leptomeningeal carcinomatosis, LCM) is believed to have increased in HER2 positive patients following the introduction in the standard therapy of specific target therapies for the HER2 receptor (in particular trastuzumab), which allow better control of systemic disease but have a poor ability to penetrate normal nerve tissue and liquor.
The therapeutic possibilities of leptomeningeal disease are therefore extremely limited, and traditional cytotoxic drugs (capecitabine and methotrexate in high doses in particular) are not very effective and / or highly toxic.
Neratinib is an irreversible inhibitor of HER2, erbB4 and EGFR, which owns an increased ability to cross a normal blood-encephalic and normal blood-liquor barrier, and it is not subject to the action of barrier proteins involving in drug-resistance (ABCB1-B2 transporters).
This drug has been shown in a single-arm phase II study (Freedman et al., 2016), a certain activity on patients with HER2 + breast carcinoma carrying brain metastases, also pre-treated with radiotherapy (8% of partial responses to MRI , with 12.5% of patients treated with 6 or more cycles).
The leptomeningeal metastases frequently coexist with the cerebral metastases, and although there are no specific data on the efficacy of neratinib on the leptomeningeal disease, on the basis of the mechanism of action, is probable that neratinib is equally effective on the leptomeningeal disease as on the parenchymal cerebral disease.

- *OBJECTIVES*
  - ➢ Primary objective:
    - 6-month OS after the diagnosis of leptomeningeal carcinomatosis
    - Intracranial-progression free survival after the diagnosis of leptomeningeal carcinomatosis
  - ➢ Secondary objectives:
    - response on whole CNS MRI based on RANO-LANO criteria
    - Improve the clinical benefit associated with stability or radiological response
    - Monitoring of treatment safety
- *INCLUSION CRITERIA*

1. Adult patients (≥ 18 years) who give informed consent.
2. HER2 + breast carcinoma with histological or cytological confirmation
3. Leptomeningeal carcinomatosis (Chamberlain et al., 2016) newly diagnosed according to the LANO criteria (based on a positivity of neoplastic cells or a high clinical and radiological suspect)
4. Possible coexistence of brain metastases (parenchymal) that have or have not received brain panirradiation or radiosurgery
5. Systemic neoplastic disease that allows a life expectancy of at least 3 months
6. Allowed the continuation of drugs for systemic disease (in particular capecitabine, trastuzumab, trastuzumab emtansine, pertuzumab, hormone therapy, docetaxel, vinorelbine).
7. Status of validity (KPS) according to the scale of Karnofsky ≥60
8. Cardiac ejection fraction> 50%
9. Results of appropriate laboratory tests, as follows:

- Hematology
• absolute neutrophil count ≥ 1.5 x 109 / L
• hemoglobin ≥ 9.0 g / dL
• platelets ≥ 100 x 109 / L
- Biochemistry
• total bilirubin <1.6 mg / dL or <of ULN.
• aspartate aminotransferase (AST; SGOT) and alanine aminotransferase (ALT; SGPT) <2.5 x ULN

• alkaline phosphatase <2.5 x ULN
• creatinine <1.5 mg / dL or creatinine clearance ≥ 50 mL / min
10. Patients who are not surgically sterile or post-menopausal must agree to abstinence or reliable contraceptive methods for the duration of the study and for 90 days following the last administration.

- *EXCLUSION CRITERIA*

1. Ongoing treatment with other anti-HER2 “small molecules” (e.g. lapatinib, pyrotinib, tucatinib), intrathecal therapy, or other investigational agents
2. Use of anti-epileptic inductors drugs
3. Pregnancy or breastfeeding
4. Any evidence of severe and uncontrollable respiratory, cardiac, hepatic or renal disease.

➢ *STUDY DESIGN*

Capecitabine is administered (750 mg/m2 twice per day for 14 days, then 7 days off)
Neratinib is administered orally (240 mg/day) without interruption, and each cycle lasts 28 days. During the 1st cycle, loperamide is administrated (as prophylaxis for diarrhea, table 1).

Table 1: changes to Loperamide dosing for the first cycle

Drug	Days 1-3	Days 3-28	Days 29+
**Loperamide**	12 mg (2 tablets x3/die)	8 mg (2 tablets x2/die)	As needed

Treatment continues until unacceptable toxicity, neurological deterioration, serious intercurrent illnesses and until patient's withdrawal. Conversely when patients displayed radiological progression, but still neurological benefit was present, neratinib plus capecitabine were continued.
Investigational product dose adjustment and/or discontinuation should be performed as described (table 2):

Table 2: Dose Reduction Levels for Investigational Product-Related Toxicity

Study Drug	Initial Dose	Dose Level -1	Dose Level -2	Dose Level -3
**Neratinib**	240 mg	160 mg	120 mg	Interruption
**Loperamide**	2 mg every 6/8 hours (3/4 tablets)	2 mg every 12 hours (2 tablets)	2 mg/die (1 tablet)	Interruption

If doses of the drug are held, study procedures for that cycle will proceed on schedule as planned, without any delay. This also applies to tumor assessments, which should continue to be done every 2 cycles, starting from the first dose of drugs until the first planned tumor assessment and then after every additional 2 cycles (± 7 days) of treatment regardless of any changes in dose or occurrence of AEs. Missed doses (any dose that is not administered within the protocol-defined administration window) will not be made up. The dose adjustment guidelines represent the minimum set of measures the Investigator must follow. However, additional measures may be taken, as necessary, for certain patients per the Investigator’s medical judgment. All dose modifications/adjustments should be documented in the patient’s source file.
Once the neratinib dose has been reduced for a patient, all subsequent cycles must be administered at that dose, unless further dose reduction is required. Dose re-escalation will only be permitted after careful evaluation of physician and evidence of this approval must be contained within the patient’s source file.
Detailed rules for dose adjustments of neratinib in case of toxicity, including the dose levels to which IP should be adjusted, are provided in Tables 3-10:

Table 3: General Toxicities Requiring Dose Adjustment

NCI CTCAE v. 4.0	Action with Neratinib
**Grade 2 adverse reaction**	
1st appearance	Hold neratinib until event resolves to Grade ≤1; then resume neratinib at the starting dose level.
2nd appearance	Hold neratinib until event resolves to Grade ≤1; then resume neratinib at 160 mg.
3rd appearance	Hold neratinib until event resolves to Grade ≤1; then resume neratinib at 120 mg.
4th appearance	Discontinue neratinib permanently.

**Grade 3 adverse reaction**	
1st appearance	Hold neratinib until event resolves to Grade ≤1; then resume neratinib at 160 mg.
2nd appearance	Hold neratinib until event resolves to Grade ≤1; then resume neratinib at 120 mg.
3rd appearance	Discontinue neratinib permanently.
**Grade 4 adverse reaction**	
1st appearance	Discontinue neratinib permanently OR if Investigator deems it to be in the patient's best interest to continue, hold neratinib until resolved to Grade ≤1; then resume neratinib at 160 mg.<br>If the event occurs again despite one dose reduction, permanently discontinued neratinib

Table 4: Gastrointestinal Toxicities Related to Diarrhea Requiring Dose Adjustment

NCI CTCAE v. 4.0	Action with Neratinib
**Grade 1 Diarrhea** (Increase of <4 stools per day over baseline)<br><br>**OR**<br><br>**Grade 2 Diarrhea** (Increase of 4-6 stools per day over baseline) lasting <5 days<br><br>**OR**<br><br>**Grade 3 Diarrhea** [Increase of ≥7 stools per day over baseline; incontinence; hospitalization indicated; severe increase in ostomy output compared to baseline limiting self-care activities of daily living (ADL)] lasting <2 days	Adjust anti-diarrheal treatment, as per the guidelines for management of diarrhea at the first onset of diarrhea. Continue neratinib at full dose.<br>Instruct patient to follow dietetic recommendations in the guidelines for management of diarrhea.<br>Fluid intake of ~2L should be maintained to avoid dehydration.<br>Once the event resolved to ≤ Grade 1 or baseline, continue loperamide as per prophylaxis guidelines.
Persisting and intolerable **Grade 2 Diarrhea** lasting >5 days despite being treated with optimal medical therapy, or associated with fever, dehydration, or **Grade 3-4 neutropenia**<br><br>OR<br><br>**Grade 3 Diarrhea** lasting > 2 days despite being treated with optimal medical therapy, or associated with fever, dehydration, or **Grade 3-4 neutropenia**<br><br>OR<br><br>Any **Grade 4 diarrhea** (Lifethreatening consequences; urgent intervention indicated)	Adjust anti-diarrheal treatment, as per the guidelines for management of diarrhea at the first onset of diarrhea.<br>Hold neratinib until recovery to ≤ Grade 1 or baseline.<br>Instruct patient to follow dietetic recommendations of the guidelines for management of diarrhea.<br>Fluid intake of ~2L should be maintained, intravenously if needed.<br>If recovery occurs:<br>≤1 week after withholding treatment, resume same dose of neratinib.<br>Within 1-4 weeks after withholding treatment, reduce neratinib dose to the next lower dose level.<br>If event occurs a 2nd time and the neratinib dose has not already been decreased, reduce neratinib dose to the next lower dose level.<br>If subsequent events occur, reduce neratinib dose to the next lower dose level.

	Once the event resolved to ≤ Grade 1 or baseline, start loperamide 4 mg with each subsequent neratinib administration.

Table 5: Pulmonary Toxicities Requiring Dose Adjustment

NCI CTCAE v. 4.0	Action with Neratinib
**Grade 2 Pneumonitis/Interstitial Lung Disease** [Symptomatic; medical intervention indicated; limiting instrumental ADL]	Hold neratinib until recovery to ≤ Grade 1 or baseline. Reduce neratinib to 160 mg or discontinue neratinib as per Investigator's best medical judgment.
**Grade ≥3 Pneumonitis/Interstitial Lung Disease** [Severe symptoms; limiting selfcare ADL; oxygen indicated]	Discontinue neratinib permanently.

Table 6: Liver Toxicity Requiring Dose Adjustment

NCI CTCAE v. 4.0	Action with Neratinib
**Grade 3 ALT (>5 – 20x ULN)**<br><br>**OR**<br><br>**Grade 3 bilirubin (>310x ULN)**	Hold neratinib until recovery to ≤ Grade 1 for patients with ALT ≤ Grade 1 at baseline OR ≤ Grade 2 for patients with Grade 2 ALT at baseline.<br>For patients with ALT ≤ Grade 1 at baseline: resume neratinib at the next lower dose level if recovery to ≤Grade 1 occurs within 4 weeks. If Grade 3 ALT or bilirubin occurs again despite one dose reduction, permanently discontinue neratinib. For patients with Grade 2 ALT at baseline due to liver metastases: contact the Sponsor for guidance on appropriate dose adjustments.
**Grade 4 ALT (>20x ULN)**<br><br>**OR**<br><br>**Grade 4 Bilirubin (>10x ULN)**	Discontinue neratinib permanently.

Table 7: Left Ventricular Ejection Fraction Toxicity Requiring Dose Adjustment

NCI CTCAE v. 4.0	Action with Neratinib
**Asymptomatic absolute decline of LVEF ≥15% from baseline OR absolute decline of LVEF ≥10% and below the lower limit of normal of 50%**	If LVEF below 40%: Hold neratinib and seek cardiology input OR continue neratinib with great caution.<br><br>Initiate monthly monitoring of LVEF<br>If while monitoring monthly LVEF remains <40%: reconsider neratinib only if appropriate and after cardiology consult.<br>If while monitoring monthly LVEF increases to ≥40%: continue neratinib, monitor LVEF every 12 weeks and consider cardiac support with input from cardiologist.<br><br>If LVEF between 40% to 50%: continue neratinib with caution and surveillance<br><br>Initiate monthly monitoring of LVEF

	If while monitoring monthly LVEF falls to <40%: Follow bullet point A instructions described above.<br>If while monitoring monthly LVEF remains ≥40%: continue neratinib, monitor LVEF every 12 weeks and consider cardiac support with input from cardiology.
**Insufficienza cardiaca sintomatica**	Neratinib should be discontinued.

If a patient has a second episode of asymptomatic decline in LVEF that meets either of the above criteria, permanently discontinue neratinib, repeat LVEF in 3 to 4 weeks and consider cardiology consult. Note that, for AEs other than asymptomatic LVEF decline, if neratinib is held for >4 weeks, the patient should be withdrawn from the treatment stage of the study. In case of asymptomatic LVEF decline, patients may resume neratinib within 1 week after LVEF recovery is documented as above, even if the timeframe exceeds 4 weeks.

Table 8: Infusion Reactions Requiring Dose Adjustment

NCI CTCAE v. 4.0	Action with Neratinib
**Dyspnea, clinically significant hypotension**	No dose adjustment required
**Severe infusion reactions**	No dose adjustment required

Table 9: Neuropathy Toxicity Requiring Dose Adjustment

NCI CTCAE v. 4.0	Action with Neratinib
**Grade 2 neuropathy**	No dose adjustment required
**Grade 3 neuropathy**	No dose adjustment required

Table 10: Hematologic Toxicity Requiring Dose Adjustmen

NCI CTCAE v. 4.0	Action with Neratinib
**ANC <1500/mm3 (1.5 × 109/L)**<br><br>**OR**<br><br>**platelet <75,000/mm3 (75 × 109/L)**	No dose adjustment required
**Grade 4 neutropenia lasting >7 days,**<br><br>**OR**<br><br>**Grade 4 febrile neutropenia**<br><br>**OR**<br><br>**Grade 3 or 4 infection with Grade 3 or 4 neutropenia (ANC < 1000)**	No dose adjustment required

Patients must be discontinued from the investigational products (but may remain in the study for long term follow up, if appropriate), under the following circumstances listed in this section and in unless otherwise agreed with the Medical Monitor:

- If the patient requires more than 2 dose reductions of IP
- If the IP is withheld due to a neratinib-related AE for >28 days. Patients who are clinically benefiting from therapy with neratinib may be resumed on therapy after 28 days if approved in advance by the Sponsor.
- Disease progression. Patients who, in the opinion of the Investigator, are continuing to benefit from neratinib despite disease progression may continue to receive neratinib
- Pregnancy
- Investigator request.

- Patient request (withdrawal of consent for treatment).

.

In the case of the following events, the patient should discontinue treatment, but should be asked to remain on study for response assessment as well as PFS if the event leading to discontinuation of treatment occurs prior to the first tumor assessment at 2 cycles, or for response assessment, PFS, and OS if the event occurs after the first tumor evaluation:

- Adverse events/toxicity
- Symptomatic deterioration
- Protocol violation
- Patient request (consent withdrawal)
- Investigator request (reasoning required)

All patients underwent physical and neurological examination, laboratory testing, ECG, echocardiogram, and CT of chest/abdomen/pelvis at baseline. Afterward, physical, and neurological examination and blood testing were performed monthly, while ECG and echocardiogram were performed every 3 months. Brain and spinal MRI with gadolinium were performed at baseline and every 2 months. CSF examination at baseline and over treatment were not mandatory to avoid further discomfort to these patients with an advanced disease. Patients underwent CT chest/abdomen/pelvis every 3 months for monitoring systemic disease.
